# Editorial: Challenges and opportunities in tumor metabolomics

**DOI:** 10.3389/fmolb.2026.1796372

**Published:** 2026-02-11

**Authors:** Lei Yin, Cheng Kong, Chunmiao Cai, Hongqi Teng, Zhengyan Chang

**Affiliations:** 1 Department of Urology, Shanghai Ninth People’s Hospital, Shanghai Jiao Tong University School of Medicine, Shanghai, China; 2 Department of Colorectal Surgery, Fudan University Shanghai Cancer Center, Shanghai, China; 3 Department of Medicine, Division of Gastroenterology and Hepatology, Mayo Clinic, Rochester, MN, United States; 4 Department of Experimental Radiation Oncology, University of Texas MD Anderson Cancer Center, Houston, TX, United States; 5 Department of Pathology, Shanghai 10th People’s Hospital, Tongji University, Shanghai, China

**Keywords:** diagnosis, metabolic reprogramming, microenvironment, therapy, tumor metabolomics

## Introduction

Tumor metabolomics has emerged as a pivotal discipline at the intersection of cancer biology, systems medicine, and translational oncology (Trefny et al., 2025). Beyond genetic and epigenetic alterations, malignant cells undergo profound metabolic reprogramming to sustain uncontrolled proliferation, survive hostile microenvironments, evade immune surveillance, and acquire invasive and metastatic capacities (Tan et al., 2026; Yin et al., 2023; Dai et al., 2025). Classical hallmarks such as the Warburg effect have now evolved into a far more complex landscape encompassing lipid remodeling, amino acid dependency, redox homeostasis, mitochondrial rewiring, and metabolite-driven epigenetic regulation (Hu et al., 2024; Yin et al., 2020; Yin et al., 2024). These metabolic adaptations are not merely consequences of oncogenic transformation, but active drivers of tumor evolution, therapeutic resistance, and immune escape.

Recent technological advances have fundamentally transformed the field. High-resolution mass spectrometry–based metabolomics, single-cell and spatial profiling technologies, and multi-omics integration strategies now enable the systematic dissection of metabolic heterogeneity within the tumor microenvironment (Liu et al., 2026). At the same time, artificial intelligence and machine learning approaches are increasingly applied to extract predictive patterns from complex metabolic datasets, accelerating biomarker discovery and clinical translation (McKenzie et al., 2026). Together, these innovations are shifting tumor metabolomics from descriptive profiling toward mechanistic understanding and precision medicine. However, significant challenges remain. Metabolic plasticity allows tumors to dynamically adapt to environmental stressors such as hypoxia, nutrient deprivation, immune pressure, and therapeutic intervention, complicating target identification and therapeutic durability (Karakousi et al., 2026). Moreover, the translation of metabolomic signatures into clinically robust diagnostics and therapeutic strategies requires rigorous validation, standardized methodologies, and integrative analytical frameworks (Long et al., 2020).

The Research Topic *“Challenges and Opportunities in Tumor Metabolomics”* brings together eleven contributions that collectively capture the conceptual, methodological, and translational dimensions of this rapidly evolving field. Zhu et al. demonstrated that the transcription factor SOX2 promotes osteosarcoma invasion and metastasis by transcriptionally activating LPCAT1, thereby reprogramming cholesterol metabolism. Their *in vitro* and *in vivo* data establish a direct link between stemness-associated transcriptional programs and lipid metabolic remodeling, highlighting cholesterol metabolism as a critical vulnerability in aggressive bone tumors. In non-small cell lung cancer, Kong et al. elucidated a glutamine metabolism–driven axis in which FGF17, activated under conditions of GLUL overexpression, sustains redox homeostasis and epithelial–mesenchymal transition through FGFR4/MEK5/ERK5/NRF2 signaling. Importantly, targeting this pathway not only suppressed invasion but also enhanced cisplatin sensitivity, illustrating how metabolic dependencies can be exploited for combinatorial therapeutic strategies. Complementing these findings, Guo et al. investigated urothelial carcinoma and revealed a strong association between HER2 positivity and elevated lactate dehydrogenase levels, linking oncogenic signaling with enhanced glycolytic activity. Their clinical data suggest that metabolic markers such as LDH may serve as accessible surrogates for aggressive, HER2-driven disease phenotypes.

A central theme emerging from this Research Topic is the role of tumor metabolism in sculpting the immune microenvironment. Dong et al. provided compelling evidence that hypoxia-induced metabolic stress in uveal melanoma promotes immune evasion through CD63-enriched exosomes. Under hypoxic conditions, tumor cells increased exosomal lactate delivery, reprogramming macrophage metabolism toward an immunosuppressive M2 phenotype and inducing CD8^+^ T-cell exhaustion. This study highlights extracellular vesicle–mediated metabolic communication as a previously underappreciated mechanism of immune suppression and identifies the hypoxia/CD63/exosomal lactate axis as a potential therapeutic target. At a systemic level, Zheng et al. integrated serum metabolomics with machine learning to predict responses to chemoimmunotherapy in advanced lung squamous cell carcinoma. By constructing a robust prognostic model based on eight metabolites, this work illustrates how metabolic signatures can inform patient stratification and therapeutic decision-making in the era of immunotherapy. Although not cancer-specific, the study by Yang et al. on myocardial infarction further reinforces the broader relevance of immunometabolic regulation. Their identification of inflammation- and immune-related hub genes through integrative bioinformatics and machine learning underscores methodological frameworks that are directly transferable to tumor metabolomics and immune-oncology research.

Beyond individual mechanisms, several contributions adopt a macroscopic view of tumor metabolomics research. Chen et al. conducted a comprehensive bibliometric analysis of spatial metabolomics in cancer, revealing rapid growth of the field and highlighting metabolic heterogeneity within the tumor microenvironment as a dominant research frontier. Their work emphasizes the transformative potential of spatially resolved metabolomics for understanding cell–cell metabolic interactions *in situ*. Similarly, Cai et al. systematically mapped global research trends in ocular tumor–associated metabolites and metabolism-related intraocular malignancies, respectively. These analyses consistently identified uveal melanoma as a central disease focus and documented a shift toward immune regulation, hypoxia, lipid metabolism, and precision oncology. Together, these studies provide valuable context for positioning mechanistic discoveries within broader scientific and clinical trajectories. In melanoma research, Bai et al. further explored global trends in resistance to BRAF and MEK inhibitors, revealing an increasing emphasis on metabolic adaptation, non-apoptotic cell death pathways, and combination therapies. These insights reinforce the concept that metabolic plasticity underlies therapeutic resistance and must be addressed to achieve durable clinical responses.

Collectively, the studies in this Research Topic highlight several key challenges and opportunities in tumor metabolomics. Methodologically, integrating metabolomics with single-cell, spatial, and computational approaches remains technically demanding but essential for capturing tumor heterogeneity. Biologically, disentangling causal metabolic drivers from adaptive consequences requires rigorous functional validation. Clinically, translating complex metabolic signatures into robust, reproducible biomarkers and therapeutic targets continues to be a major hurdle. By bridging mechanistic discovery with computational innovation and translational relevance, this Research Topic underscores tumor metabolomics as a dynamic and integrative field poised to reshape cancer diagnosis and therapy. Continued interdisciplinary collaboration and methodological innovation will be critical to fully harness the clinical potential of tumor metabolomics in the years ahead.

## References

[B1] Dai Y. Guo S. Pan Y. Castignani C. Montierth M. D. Van Loo P. (2025). A guide to transcriptomic deconvolution in cancer. Nat. Rev. Cancer. 10.1038/s41568-025-00886-9 PMC7618897 41331516

[B2] Hu Y. Liu W. Fang W. Dong Y. Zhang H. Luo Q. (2024). Tumor energy metabolism: implications for therapeutic targets. Mol. Biomed. 5 (1), 63. 10.1186/s43556-024-00229-4 39609317 PMC11604893

[B3] Karakousi T. Cristaldi V. Lopes de Oliveira M. L. Delclaux I. Besson N. R. Geraldo L. H. (2026). IFNgamma-dependent metabolic reprogramming restrains an immature, pro-metastatic lymphatic state in melanoma. Cancer Cell. 10.1016/j.ccell.2025.12.019 PMC12930414 41576931

[B4] Liu Y. Dai Y. Wang L. (2026). Spatial omics at the forefront: emerging technologies, analytical innovations, and clinical applications. Cancer Cell 44 (1), 24–49. 10.1016/j.ccell.2025.12.009 41478277 PMC12867001

[B5] Long N. P. Nghi T. D. Kang Y. P. Anh N. H. Kim H. M. Park S. K. (2020). Toward a standardized strategy of clinical metabolomics for the advancement of precision medicine. Metabolites 10 (2). 10.3390/metabo10020051 32013105 PMC7074059

[B6] McKenzie M. Irac S. E. Chen Z. Moradi A. Jenner A. Nguyen Q. (2026). Integrative spatial omics and artificial intelligence: transforming cancer research with omics data and AI. Semin. Cancer Biol. 119, 65–82. 10.1016/j.semcancer.2026.01.002 41520911

[B7] Tan Y. Xue R. Pan Y. He Z. Hu X. Li Y. (2026). Cancer cachexia: molecular basis and therapeutic advances. Signal Transduct. Target Ther. 11 (1), 16. 10.1038/s41392-025-02331-7 41526343 PMC12795897

[B8] Trefny M. P. Kroemer G. Zitvogel L. Kobold S. (2025). Metabolites as agents and targets for cancer immunotherapy. Nat. Rev. Drug Discov. 24 (10), 764–784. 10.1038/s41573-025-01227-z 40571788

[B9] Yin L. Li W. Xu A. Shi H. Wang K. Yang H. (2020). SH3BGRL2 inhibits growth and metastasis in clear cell renal cell carcinoma via activating hippo/TEAD1-Twist1 pathway. EBioMedicine 51, 102596. 10.1016/j.ebiom.2019.12.005 31911271 PMC7000347

[B10] Yin L. Li W. Chen X. Wang R. Zhang T. Meng J. (2023). HOOK1 inhibits the progression of renal cell carcinoma via TGF-beta and TNFSF13B/VEGF-A axis. Adv. Sci. (Weinh) 10 (17), e2206955. 10.1002/advs.202206955 37085921 PMC10265082

[B11] Yin L. Mao L. Yin R. Lv C. Shi X. Yue C. (2024). ACE loss drives renal cell carcinoma growth and invasion by modulating AKT-FOXO1. Biologics 18, 397–412. 10.2147/BTT.S485178 39717370 PMC11665188

